# Reappraisal capacity is unrelated to depressive and anxiety symptoms

**DOI:** 10.1038/s41598-023-33917-2

**Published:** 2023-05-03

**Authors:** Jack L. Andrews, Tim Dalgleish, Jason Stretton, Susanne Schweizer

**Affiliations:** 1 School of Psychology, UNSW, Sydney, Australia; 2 MRC Cognition and Brain Sciences Unit, University of Cambridge, Cambridge, UK; 3 Department of Psychology, University of Cambridge, Cambridge, UK

**Keywords:** Psychology, Human behaviour, Depression

## Abstract

Research suggests affective symptoms are associated with reduced habitual use of reappraisal as an emotion regulation strategy in individuals with mental health problems. Less is known, however, about whether mental health problems are related to reduced reappraisal capacity per se. The current study investigates this question using a film-based emotion regulation task that required participants to use reappraisal to downregulate their emotional response to highly evocative real-life film footage. We pooled data (*N* = 512, age: 18–89 years, 54% female) from 6 independent studies using this task. In contrast to our predictions, symptoms of depression and anxiety were unrelated to self-reported negative affect after reappraisal or to emotional reactivity to negative films. Implications for the measurement of reappraisal as well as future directions for research in the field of emotion regulation are discussed.

## Introduction

Emotions are a fundamental driver of human cognition and behavior. Yet, they frequently require regulation to meet everyday goal demands. Emotion regulation refers to the ability to change one’s emotional state by modifying one or more of a set of sequential processes: identification, selection, and implementation of appropriate regulatory strategies^1^.

This capacity, to successfully identify, select and implement the most appropriate regulatory strategy, varies across individuals and has been proposed to be compromised in those experiencing mental health problems^2^. While much research has mapped the association between habitual use of specific emotion regulation strategies and mental health problems^3^, comparatively less is known about variance in the capacity to implement specific emotion regulation strategies and mental health^4^. Here we combine data from a series of studies to explore the association between the capacity to regulate emotions using one such strategy—cognitive reappraisal—and symptoms of depression and anxiety.

Cognitive reappraisal requires the reinterpretation of an emotion-eliciting event in order to alter its significance, thereby down- or up-regulating the affective response to the event^1^. Individuals with higher trait usage of cognitive reappraisal have been found to report fewer depressive symptoms and negative emotions, relative to individuals with lower trait cognitive reappraisal^3^. Further, studies have shown that down-regulation of negative affect is possible when individuals are instructed to implement cognitive reappraisal in laboratory behavioural and functional Magnetic Resonance Imaging (MRI) tasks^1,4^. However, it remains unclear whether individuals’ capacity to implement reappraisal is related to mental health, with research reporting mixed findings^4,5^.

Despite the mixed empirical findings from typically small samples, theories of depressive and anxiety disorders propose that difficulties in regulating emotions are critical to both the onset and maintenance of emotional disorders^6–9^. In particular, we focus on reappraisal capacity, given previous findings showing its ability to mediate the link between stress and depressive symptoms^10^, to modify physiological^11–13^ and affective^14–16^ responses to negative emotions. Further, reappraisal is a central component of cognitive behavioral therapy for depression^17^ highlighting it’s hypothesized role in mental health disorders. In the current study, we therefore sought to examine the association between reappraisal capacity and symptoms of depression and anxiety in a well-powered sample. Given the role of reappraisal in reducing negative emotions and affect in response to negative stimuli and stressors^12,18^, the current study operationalized reappraisal capacity as individuals’ ability to down-regulate emotional responses to highly evocative negative film footage by thinking about the content of the footage differently. Self-reported distress in the regulation condition was compared to trials in which participants simply watched negative footage without trying to down-regulate their emotional response. Previous research using variations of this task (e.g. using different films or images) has found no significant associations between performance on this task and mental health outcomes^4,5,19,20^.

These results may be partially accounted for by methodological and demographical factors. First, the relatively small samples commonly used in experimental psychopathology research may have obscured or inflated effects of mental health on emotion regulation capacity observed in previous research. Combining evidence from a series of studies that used the same task then can help elucidate the association between mental health problems and emotion regulation capacity by increasing the statistical power to detect an effect. The present study pooled data from 512 participants who completed the same emotion regulation task and a measure of symptoms of depression or anxiety. Second, the differences may be partially accounted for by developmental differences in emotion regulation capacity. Previous research as shown age-related variance in habitual use of emotion regulation strategies^21^ and the brain areas involved in successful emotion regulation^22,23^. The wide age range (18–89 years) included in the current study allowed us to investigate age-related variation in emotion regulation and emotional reactivity. Third, while gender differences in habitual use of emotion regulation have been reported^3^, less is known about gender differences in emotion regulation capacity. We therefore additionally explored gender differences in emotion regulation capacity and their association with anxiety and depressive symptoms. Finally, the task design allowed us to additionally investigate the association between emotional reactivity and symptoms of depression and anxiety, by comparing distress ratings to neutral film clips to negative films that participants were instructed to watch while allowing their emotions to arise freely. Depressive symptoms have been associated with reduced emotional reactivity in line with the emotion context insensitivity hypothesis^24^.

In sum, the current study examined the following hypotheses: In line with theoretical models of the role of reduced emotion regulation capacity in individuals with mental health problems, we predicted that reappraisal capacity (i.e., the capacity to reduce distress in the regulation relative to the negative watch condition) would be inversely associated with individuals’ levels of depression and anxiety symptoms (*hypothesis 1*). Second, we predicted that emotional reactivity (distress reported in the negative watch condition compared to the neutral watch condition) would decrease as a function of depressive and anxiety symptoms (*hypothesis 2*). Finally, we explored whether age or gender accounted for variance in the association between emotion regulation capacity and symptoms of depression and anxiety.

## Results

### Relationship between reappraisal capacity and reactivity and mental health

**Figure 1:**
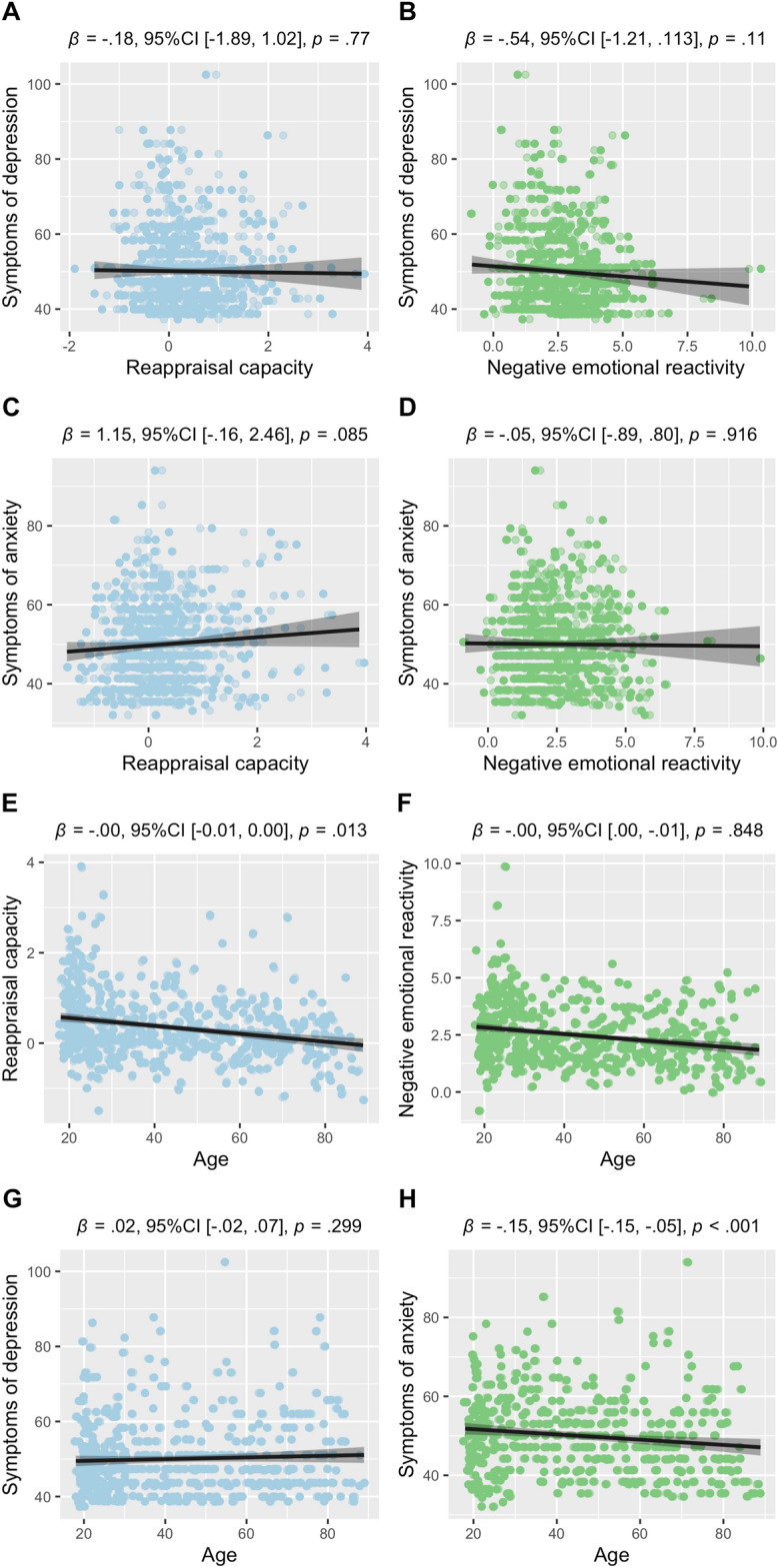
Associations between task performance, mental health and age. Panels (**A**, **B**) show associations between emotion regulation (reappraisal capacity) and reactivity and depression. Panels (**C**, **D**) show associations between emotion regulation and reactivity and anxiety. Panels (**E**, **F**) show associations between regulation and reactivity and age. Raw scores are plotted, whilst beta values from each main effects mixed model are displayed above each graph. Regulatory capacity was computed by subtracting participants’ self-reported distress during the watch negative condition from distress reported in the regulate negative condition (i.e., higher regulation scores represent greater regulation ability. Emotional reactivity index by subtracting participants’ self-reported distress during the watch negative condition from the distress reported in the watch neutral condition. Depression and anxiety symptoms are represented by T scores, given the discrepancy in measures used across samples. Higher scores index greater regulation capacity, emotional reactivity and depression and anxiety symptoms.

Descriptive statistics for all variables can be found in Table 1, and correlation plots are presented in Fig. 1.

**Table 1:** Descriptive statistics for each variable of interest, for the total sample, and each sub-sample.

Sample	Mean (standard deviation) and range						
	Total	1^20^	2^23^	3^45^	4^44^	5	6
N	512	98	256	48	29	48	33
Age	43.74 (20.40)	52.14 (17.45)	53.02 (18.43)	20.10 (.69)	23.28 (2.43)	24.96 (4.42)	26.45 (8.45)
Reappraisal	0.35 (0.73)	0.63 (0.77)	0.06 (.46)	0.66 (0.75)	0.70 (0.82)	0.65 (0.97)	0.57 (0.89)
	–1.50–3.88	–1.20–2.80	–1.25–1.65	–0.40–2.50	–0.80–2.30	–1.50–3.88	–0.50–3.30
Reactivity	2.48 (1.30)	3.37 (1.04)	1.72 (0.70)	2.39 (0.94)	2.64 (1.05)	4.27 (1.57)	3.21 (1.18)
	–0.83–9.88	0.10–5.60	–0.04–4.31	–0.83–4.00	0.60–4.90	1.00–9.88	1.10–6.20
Depression	50.06 (10.07)	49.93 (10.13)	50.05 (10.20)	49.72 (9.70)	47.94 (6.80)	49.97 (9.58)	53.03 (12.34)
	37.31–102.47	38.68–84.11	39.97–102.47	37.31–81.33	38.85–71.80	38.85–79.71	38.85–86.3
Anxiety	50.01 (9.94)	50.21 (10.14)	49.85 (9.94)	49.91 (10.15)	N/A	47.05 (10.65)	55.01 (5.69)
	32.07–94.01	34.62–81.48	35.42–94.01	35.28–75.22		32.07–78.35	43.09–66.23

The depression and anxiety scores are t-scores based on the scaled scores of the Hospital and Anxiety Scale for samples 1 and 2; Mood and Feelings Questionnaire (depression) and Spielberger State and Trait Anxiety Inventory (anxiety) for sample 3; Beck Depression Inventory-II (depression) for samples 4–6 and the state version of the Spielberger State and Trait Anxiety Inventory (anxiety) for samples 5–6. State anxiety symptoms were not measured in sample 4. Samples 5 and 6 are the two unpublished datasets. Reappraisal capacity was computed by subtracting participants’ self-reported distress during the watch negative condition from distress reported in the regulate negative condition (i.e., higher regulation scores represent greater regulation ability) in our task (see “Methods”). Emotional reactivity index by subtracting participants’ self-reported distress during the watch negative condition from the distress reported in the watch neutral condition in our task. We reversed these scores such that higher reactivity scores index greater negative emotional reactivity.

In contrast with our first hypothesis neither symptoms of depression (β = − 0.18, *p* = 0.77) nor anxiety (β = 1.15, *p* = 0.085) were significantly associated with reappraisal capacity. The absent main effect in both models was supported by Bayes factors (depression: BF_10_ = 0.046; anxiety: BF_10_ = 0.154). Further, in contrast with our predictions neither symptoms of depression (β = − 0.54, *p* = 0.113) nor anxiety (β = − 0.05, *p* = 0.916) were associated with self-reported emotional reactivity to negative film clips. Again, the frequentist results were supported by a null Bayes factor (depression: BF_10_ = 0.20; anxiety: BF_10_ = 0.046). See Table 2 for model outputs.

**Table 2:** Models predicting mental health symptoms (depression and anxiety), with reappraisal capacity and reactivity.

Predictors	Depression			Anxiety		
	Estimates	CI	p	Estimates	CI	p
Reappraisal model						
(Intercept)	50.12	49.16–51.09	** < 0.001**	49.68	47.77–51.59	** < 0.001**
Reappraisal	− 0.18	− 1.38–1.02	0.770	1.15	− 0.16–2.46	0.085
Observations	512			480		
Marginal R^2^/conditional R^2^	0.000/NA			0.007/0.035		
Reactivity model						
(Intercept)	51.41	49.53–53.28	** < 0.001**	50.34	47.34–53.35	** < 0.001**
Reactivity	− 0.54	− 1.21–0.13	0.113	− 0.05	− 0.89–0.80	0.916
Observations	512			480		
Marginal R^2^/conditional R^2^	0.005/NA			0.000/0.026		

Significant values are in bold.

The marginal R^2^ value indicates the variance explained by the fixed effects alone, and the condition R^2^ represents the variance explained by the entire model (including the random effect of participant, nested by sample).

### Age effects

A model predicting reappraisal capacity with age revealed a significant main effect of age (β = − 0.00, *p* = 0.013), with reappraisal capacity decreasing with age. However, this effect of age on regulation was not supported by the Bayes factor in favour of the null hypothesis (BF_10_ = 0.80). A model predicting reactivity with age showed no significant effect of age (β = 0.00, *p* = 0.848), supported by a null Bayes factor (BF_10_ = 0.045). See Table 3 for model outputs.

**Table 3:** Models predicting reappraisal capacity and reactivity with age.

Predictors	Reappraisal			Reactivity		
	Estimates	CI	p	Estimates	CI	p
(Intercept)	0.68	0.48–0.89	** < 0.001**	2.91	2.23–3.59	** < 0.001**
Age	− 0.00	− 0.01–0.00	**0.013**	0.00	0.00–0.01	0.848
Observations	512			512		
Marginal R^2^/conditional R^2^	0.018/0.089			0.000/0.415		

Significant values are in bold.

The marginal R^2^ value indicates the variance explained by the fixed effects alone, and the condition R^2^ represents the variance explained by the entire model (including the random effect of participant, nested by sample).

Additionally, models predicting symptoms of depression with an interaction between regulation and age (β = − 0.03, *p* = 0.305; BF_10_ = 0.0002) and reactivity and age (β = 0.003, *p* = 0.982; BF_10_ = 0.0004) revealed non-significant effects, both supported by null Bayes factors. The same was observed for models predicting symptoms of anxiety with an interaction between regulation and age (β = 0.01, *p* = 0.872; BF_10_ = 0.155) and reactivity and age (β = 0.001, *p* = 0.497; BF_10_ = 0.077) revealed non-significant effects, also supported by nulls Bayes factors. See Table 4 for model outputs.

**Table 4:** Models predicting mental health symptoms (depression and anxiety), with interactions between reappraisal capacity and age, and reactivity and age, respectively.

Predictors	Depression			Anxiety		
	Estimates	CI	p	Estimates	CI	p
Reappraisal model						
(Intercept)	48.72	46.36–51.08	** < 0.001**	53.38	50.17–56.59	** < 0.001**
Reappraisal	1.19	− 1.44–3.82	0.375	0.70	− 2.07–3.47	0.619
Age	0.03	− 0.02–0.08	0.208	− 0.10	− 0.15 to − 0.04	** < 0.001**
Regulation × age	− 0.03	− 0.09–0.03	0.305	0.01	− 0.06–0.07	0.872
Observations	512			480		
Marginal R^2^/conditional R^2^	0.004/NA			0.045/0.099		
Reactivity model						
(Intercept)	50.54	46.12–54.96	** < 0.001**	55.28	50.13–60.43	** < 0.001**
Reactivity	− 0.47	− 1.95–1.01	0.532	− 0.57	− 2.22–1.08	0.499
Age	0.02	− 0.07–0.11	0.715	− 0.12	− 0.22 to − 0.03	**0.010**
Reactivity × age	− 0.003	− 0.03–0.03	0.982	0.01	− 0.02–0.05	0.497
Observations	512			480		
Marginal R^2^/conditional R^2^	0.006/NA			0.037/0.081		

Significant values are in bold.

The marginal R^2^ value indicates the variance explained by the fixed effects alone, and the condition R^2^ represents the variance explained by the entire model (including the random effect of participant, nested by sample).

### Gender effects

Descriptive statistics for each variable, by gender, can be found in Supplementary Table 1. Models investigating the effect of gender on reappraisal capacity and reactivity, also revealed no significant main effect of gender on reappraisal capacity (β = 0.03, *p* = 0.566; BF_10_ = 0.044) or reactivity (β = 0.14, *p* = 0.093; BF_10_ = 0.049), both supported by null Bayes factors (see Supplementary Table 2).

Additionally, models predicting mental health symptoms with an interaction between reappraisal capacity and gender, revealed non-significant effects for depression (β = − 0.04, *p* = 0.975; BF_10_ = 0.00009) and anxiety (β = − 1.40, *p* = 0.265*;* BF_10_ = 0.0008), both supported by null Bayes factors (see Supplementary Table 3). Further, models predicting mental health symptoms with an interaction between reactivity and gender, revealed non-significant effects for depression (β = 0.67, *p* = 0.335; BF_10_ = 0.0005) and anxiety (β = − 0.71, *p* = 0.314*;* BF_10_ = 0.0002), both supported by null Bayes factors (see Supplementary Table 4).

## Discussion

Mental health problems, especially emotional disorders^25^, are characterised by dysregulated affect. Individuals living with mental health problems also report difficulty regulating their emotions^26^ and less habitual use of reappraisal as an emotion regulation strategy^3^. Yet, evidence for the role of reappraisal capacity in mental health is sparse. In the present study we investigated the association between an experimental task-based measure of reappraisal capacity and symptoms of depression and anxiety. We showed that reappraisal capacity and emotional reactivity were not significantly associated with self-reported depressive and anxiety symptoms.

Our findings raise the possibility that behavioural measures of reappraisal capacity are not sufficiently sensitive to deficits in reappraisal reported by individuals suffering from mental health problems. Alternatively, individuals suffering from mental health problems may not show deficits in deploying specific emotion regulation strategies when instructed to do so. However, it is important to note that like all experimental task-based investigations of emotion regulation our results are restricted by context. That is, reappraising one’s emotions to aversive films in a laboratory setting may not provide a good proxy for real world instances that require the deployment of adaptive emotion regulation strategies.

The lack of association between reappraisal capacity and symptoms of depression and anxiety observed in the current study is noteworthy. This is because in contrast with most previous work studying emotion regulation capacity using experimental tasks^5^, this study was well-powered to detect an effect of emotion regulation on mental health. A possible account for the lack of association between mental health and reappraisal capacity is that instead of deficits in reappraisal capacity per se, individuals with mental health problems struggle to flexibly deploy situationally-adaptive emotion regulation strategies. In support of this account, the ability to flexibly regulate one’s emotions in line with changes in the external environment has been associated with good mental health^27,28^. Emotion regulation flexibility is adaptive insofar as it increases the ability for individuals to meet their personal goals in emotion eliciting situations^27^. Critically, in the context of mental health reappraisal capacity, the habitual tendency to use reappraisal and reappraisal quality (see reappraisal inventiveness below) may interact to influence mental health^29^. One candidate cognitive process that underpins emotion regulation flexibility and capacity is affective control. Affective control is the capacity to engage and disengage with emotional information depending on situational demands^30^. It has been proposed that affective control facilitates the flexible deployment of adaptive emotion regulation strategies at each stage of the regulatory process (identification, selection and implementation) through, strategy stopping or switching, strategy maintenance and monitoring^31^. Assessing affective control in the lab then may constitute a better proxy of emotion regulation difficulties experienced in everyday life.

Another index of reappraisal capacity is *reappraisal inventiveness*^32^, which taps into individuals’ capacity to generate different reappraisals of negative situations. Novel behavioral paradigms (e.g.,^33^) have emerged that may provide alternative behavioral measurements of reappraisal capacity. Research shows reappraisal inventiveness is associated with differential brain activation during successful reappraisal and it has been related to depressive symptoms^34–36^. However, reappraisal inventiveness has also been shown to be separable from reappraisal effectiveness^33^. Reappraisal inventiveness is therefore unlikely to fully capture theorized clinical differences in reappraisal capacity in individuals with emotional disorders.

To unpack the relation between reappraisal capacity as measured in the current study and mental health further we also examined any effects of age and gender. We found no effect of gender on our results. We did find age-related variance in emotion regulation capacity and reactivity, with observed declines in regulation ability and declines in emotional reactivity as individuals became older. These results sit in contrast to evidence that points to increased positivity in older age, and are more in line with broader ageing literature showing declines across various other domains of functioning^37^. However, recent work which took a more nuanced approach, by assessing positive and negative affect on distinct scales, found increasing positive and negative affect with age, indicating a general increase in emotionality with age. This suggests that assessing distress with one continuous scale with positive and negative at the polar ends may not be accurately capturing positive and negative affect^38^.

The current study should be considered in the context of a number of limitations. Firstly, we did not observe any gender differences in our mental health outcomes. This is surprising given that numerous previous studies have found that females report significantly higher mental health problems, relative to males. The lack of an association between gender and mental health outcomes may be due to a self-selection bias in the samples we draw on. Individuals who are willing to, or seek out the opportunity to, participate in laboratory studies related to mental health are unlikely to be population representative. Second, we did not have questionnaire-based data on individual’s emotion regulation abilities to dissociate our task-based findings from self-report. The current study also did not assess individuals’ beliefs about the controllability of emotions and their ability to control them. Beliefs about reappraisal ability have been shown to be predictive of regulatory success^39^ A further limitation is that the study design may have introduced demand effects by instructing participants to downregulate their emotions in the reappraisal condition. However, this issue is not specific to the current study and was partly mitigated by measuring participants’ emotional response rather than their self-perceived ability to “down-regulate their emotions”. That is, reappraisal was operationalized as a difference score in emotion response to watch vs. regulate trials. However, it is still possible that demand effects influenced our findings, given that participants were told that reappraisal can be used to reduce intensive negative feelings. In addition, it is worth noting the variety of possible reappraisals individuals can deploy, and our instructions may have biased participants towards particular types of reappraisal, specifically reappraisal inventiveness. Future work should consider the association between mental health and different types of reappraisal. Collectively, these points may indicate an unmeasured bias in the present sample which has contributed to these null findings. Future work should seek to replicate these findings in larger, more diverse and population representative samples.

Moving forward, the development of more accurate and ecologically valid experimental measures of emotion regulation should be explored, to better index the relationship between self-reported emotion regulation difficulties and mental health. However, it is possible that experimental measures are unable to accurately track the dynamic and changing nature of emotions, and how they are regulated, in real world settings^40,41^.

One approach that has shown promise is the use of experiencing sampling, in which participants are asked via their mobile phone to report on their feelings, and how they were choosing to regulate their emotions in the present moment. Current findings in this area have shown important associations between the degree to which individuals vary their emotion regulation strategies and negative affect^42,43^. Individuals who use a diverse profile of emotion regulation strategies have been shown to experience lower anxiety and greater positivity compared to individuals who more frequently suppressed their emotions^42,43^.

In summary, the current findings suggest that emotion regulation capacity per se is not reduced in those experiencing symptoms of mental health problems. Efforts should be invested in the development of experimental paradigms testing emotion regulation flexibility as well as experience sampling methodology capturing emotion regulation flexibility in the real world.

## Methods

### Participants

We report how we determined our sample size, all data exclusions, all manipulations, and all measures in the study. Data from 512 participants’ (mean age = 43.74, SD = 20.40) were pooled from four published studies^20,38,44,45^ and from two unpublished datasets. Our sample size was therefore determined based on the available data within each study. In all cases the emotion regulation task and self-report measures of depression and anxiety symptoms were administered. All samples were non-clinical populations. Two hundred and seventy-four (54%) participants self-identified as female, and 46% as male. Three hundred and ninety-two participants provided data on their education level, of whom 6% reported no education, 17% reported education to UK GCSE level (school age 16), 10% to UK A level (school age 18) and 67% to university degree level. Anxiety symptoms were not available in one of the unpublished datasets (*N* = 32) and are therefore are not included in analyses involving this measure. The study protocol for each study was approved by the Cambridgeshire 2 Ethics Committee (10/H0308/50; CPMS ID 10120). Written informed consent was obtained from all participants. All experiments were performed in accordance with relevant guidelines and regulations.

### Measures

#### Symptoms of depression and anxiety

Two studies^20,38^ administered the 14-item Hospital Anxiety and Depression Scale (HADS^46^) to assess symptoms of depression and anxiety. Two other studies^44,47^ and the unpublished datasets assessed symptoms of depression with the Beck Depression Inventory (BDI-II^48^) and one study^45^ used the Mood and Feelings Questionnaire (MFQ^49^). One unpublished dataset and one study^47^ assessed anxiety with the State-Trait Anxiety Inventory (STAI^50^). One study^44^ did not assess state anxiety. All measures have shown good psychometric properties^46,50,51^. Given the use of different scales, we converted raw scores into T scores^52^.

#### Emotion regulation task

The task presented participants with 30-s film clips that were viewed in three different conditions: (1) in the NEUTRAL WATCH condition, affectively-neutral footage (e.g. a weather forecast) was viewed with instructions not to engage in any emotion regulation; (2) the NEGATIVE WATCH condition in which the instructions were identical to the neutral watch condition, but participants were now presented with aversive film clip footage (e.g., war documentaries); and (3) the REGULATE condition, in which aversive footage was viewed and participants were asked to try to down-regulate their distress to the footage by thinking about the content of the films differently (i.e., engaging in reappraisal). Specifically, they were asked to “Try and change the way you feel about the film clip by changing the way you think about its content.” They were given a few real-world examples of reappraisal (for a sample scenario provided to participants see Supplemental materials).

**Figure 2:**
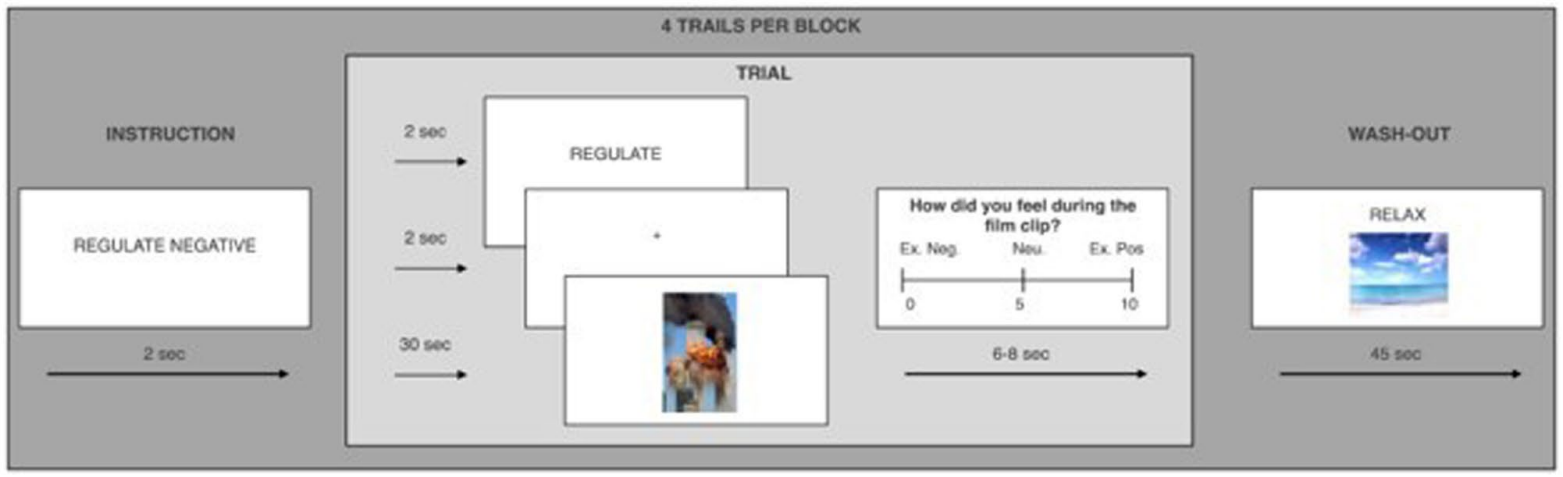
Emotion regulation task. A sample trial of the emotion regulation task. Participants viewed a series of 30 s film clips. In the neutral ‘watch’ condition, participants were asked to simply watch the films and allow their emotions to arise naturally. In the negative watch condition, participants were presented with aversive film clip footage. In the ‘regulate’ condition (shown in the figure), participants were asked to downregulate their emotions to a negative film reappraising the way they thought about the film’s content. After each film clip, participants rated how they felt during the film on a scale from ‘0 = Extremely negative’ to ‘10 = Extremely positive’. After each positive or negative film clip, participants watched a brief washout clip to return their mood to normal. The figure is reproduced from^20^.

After each film clip, participants were asked to rate the distress they experienced whilst viewing the film, on a Likert scale ranging from 0 (extremely negative) to 10 (extremely positive) (see Fig. 2). Before completing the task, participants completed a number of practice trials. In one study^23^ emotional experience was rated on two separate scales, one ranging from neutral to extremely positive, the other from neutral to extremely negative. For comparability these scores were combined to one indicator of distress. The films included in the negative WATCH and REGULATE conditions were counterbalanced across participants.

Reappraisal capacity was computed by subtracting participants’ self-reported distress during the watch negative condition from distress reported in the regulate negative condition (i.e., higher regulation scores represent greater regulation ability). In addition to regulation, we computed an emotional reactivity index by subtracting participants’ self-reported distress during the watch negative condition from the distress reported in the watch neutral condition. We reversed these scores such that higher reactivity scores index greater negative emotional reactivity.

Across studies the task showed a large reactivity effect, *d* = 3.6 measured as the effect of condition (neutral, negative watch) in a mixed effects model with Study ID as random effect. Across the studies there was a small regulation effect, *d* = 0.23 measured as the effect of condition (negative watch, negative regulate) in a mixed effects model with Study ID as random effect.

### Statistical analysis

All analyses were conducted in R (version 4.0.2). Data from each of the six independent studies were combined. Linear mixed effects models predicting depression and anxiety were used to examine main and interaction effects between age and emotion regulation or reactivity, and gender and emotion regulation or reactivity. Linear mixed effects models were also used to investigate the main effects of age and gender on emotion regulation and reactivity. A random intercept was specified in each model with participant ID nested by dataset, to account for the clustered nature of the data. Bayes factors were computed for main and interaction effects using the Bayesian Information Criterion (BIC^53^). This study was not preregistered, data and analysis code can be obtained by contacting the authors.

## Supplementary Information

Supplementary Tables.

## Data Availability

The data is available on reasonable request from the corresponding author.
