# Supplementary material for: Reappraisal capacity is unrelated to depressive and anxiety symptoms
Source: Sci Rep. 2023 May 3;13:7189. doi: 10.1038/s41598-023-33917-2 (PMC10156669; doi:10.1038/s41598-023-33917-2)
Supplement: Supplementary file 1 — Supplementary Tables. [file 41598_2023_33917_MOESM1_ESM.docx]

**Supplementary material for “Reappraisal capacity is unrelated to depressive and anxiety symptoms”**

|  | **Male** | **Female** |
| --- | --- | --- |
|  | *M* (*SD*) | *M* (*SD*) |
| N | 238 | 274 |
| Age | 44.71 | 42.10 |
| Reappraisal | 0.32 (0.71) | 0.37 (0.74) |
| Reactivity | 2.38 (1.20) | 2.58 (1.38) |
| Depression | 50.11 (10.29) | 50.02 (9.89) |
| Anxiety | 50.19 (10.28) | 49.85 (9.64) |

***Table S1.*** *Descriptive statistics for the total sample, for each variable of interest, by gender.*

|  | **Reappraisal** | | | **Reactivity** | | |
| --- | --- | --- | --- | --- | --- | --- |
| *Predictors* | *Estimates* | *CI* | *p* | *Estimates* | *CI* | *p* |
| (Intercept) | 0.48 | 0.21 – 0.74 | **<0.001** | 2.71 | 2.01 – 3.41 | **<0.001** |
| Gender | 0.03 | 0.08 – 0.15 | 0.566 | 0.14 | 0.02 – 0.31 | 0.093 |
| Observations | 512 | | | 512 | | |
| Marginal R^2^ / Conditional R^2^ | 0.001 / 0.098 | | | 0.003 / 0.415 | | |

***Table S2.*** *A model predicting reappraisal capacity with gender revealed no significant main effect of gender* (β=.03, *p*=.566), *supported by a null Bayes factor (BF_10_ = 0.044). A model predicting reactivity with gender revealed no significant main effect of gender* (β=.14, *p*=.093), *supported by a large Bayes factor (BF_10_ = 0.049).* The marginal R^2^ value indicates the variance explained by the fixed effects alone, and the condition R^2^ represents the variance explained by the entire model (including the random effect of participant, nested by sample).

|  | **Depression** | | | **Anxiety** | | |
| --- | --- | --- | --- | --- | --- | --- |
| *Predictors* | *Estimates* | *CI* | *p* | *Estimates* | *CI* | *p* |
| (Intercept) | 50.22 | 47.11 – 53.33 | **<0.001** | 49.56 | 46.00 – 53.12 | **<0.001** |
| Reappraisal | -0.12 | -4.05 – 3.81 | 0.953 | 3.32 | -0.70 – 7.33 | 0.105 |
| Gender | -0.06 | -2.00 – 1.87 | 0.948 | 0.09 | -1.84 – 2.02 | 0.929 |
| Reappraisal x Gender | -0.04 | -2.45 – 2.37 | 0.975 | -1.40 | -3.86 – 1.06 | 0.265 |
| Observations | 512 | | | 480 | | |
| Marginal R^2^ / Conditional R^2^ | 0.000 / NA | | | 0.010 / 0.041 | | |

***Table S3.*** *A model predicting symptoms of depression with an interaction between reappraisal capacity and gender revealed no significant interaction* (β=-.04, *p*=.975)*, supported by a null Bayes factor (BF_10_ = 0.00009). A model predicting symptoms of anxiety with an interaction between reappraisal capacity and gender revealed no significant interaction* (β=-1.40, *p*=.265)*, supported by a null Bayes factor (BF_10_ = 0.0008).* The marginal R^2^ value indicates the variance explained by the fixed effects alone, and the condition R^2^ represents the variance explained by the entire model (including the random effect of participant, nested by sample).

|  | **Depression** | | | **Anxiety** | | |
| --- | --- | --- | --- | --- | --- | --- |
| *Predictors* | *Estimates* | *CI* | *p* | *Estimates* | *CI* | *p* |
| (Intercept) | 54.00 | 47.79 – 60.22 | **<0.001** | 48.08 | 41.51 – 54.65 | **<0.001** |
| Reactivity | -1.62 | -3.92 – 0.68 | 0.167 | 1.10 | -1.26 – 3.45 | 0.361 |
| Gender | -1.64 | -5.44 – 2.16 | 0.398 | 1.41 | -2.40 – 5.22 | 0.468 |
| Reactivity x Gender | 0.67 | -0.70 – 2.05 | 0.335 | -0.71 | -2.09 – 0.67 | 0.314 |
| Observations | 512 | | | 480 | | |
| Marginal R^2^ / Conditional R^2^ | 0.007 / NA | | | 0.002 / 0.027 | | |

***Table S4.*** *A model predicting symptoms of depression with an interaction between reactivity and gender revealed no significant interaction* (β=.67, *p*=.335)*, supported by a null Bayes factor (BF_10_ = 0.0005). A model predicting symptoms of anxiety with an interaction between reactivity and gender revealed no significant interaction* (β=-.71, *p*=.314)*, supported by a null Bayes factor (BF_10_ = 0.0002).* The marginal R^2^ value indicates the variance explained by the fixed effects alone, and the condition R^2^ represents the variance explained by the entire model (including the random effect of participant, nested by sample).

**Example scenario of real-world reappraisal**

This example was provided to participants are part of the reappraisal instructions. “Imaging returning to your parked car and notice a thin long scratch all down its side. You may feel immediately angry assuming someone scratched your car intentionally, or you could try to change the way you feel about the situation by assuming it was an accident. Reinterpreting the situation would still leave you with negative feelings about the scratch, but they would likely be less intensive than if you thought the scratch was intentional.”
